# Cortico-muscular coupling and motor performance are modulated by 20 Hz transcranial alternating current stimulation (tACS) in Parkinson’s disease

**DOI:** 10.3389/fnhum.2013.00928

**Published:** 2014-01-16

**Authors:** Vanessa Krause, Claudia Wach, Martin Südmeyer, Stefano Ferrea, Alfons Schnitzler, Bettina Pollok

**Affiliations:** ^1^Institute of Clinical Neuroscience and Medical Psychology, Medical Faculty, Heinrich-Heine-University DuesseldorfDuesseldorf, Germany; ^2^Department of Neurology, Medical Faculty, University Hospital DuesseldorfDuesseldorf, Germany

**Keywords:** magnetoencephalography (MEG), Parkinson’s disease, motor control, cortico-muscular coupling (CMC), transcranial alternating current stimulation (tACS), primary motor cortex

## Abstract

Parkinson’s disease (PD) is associated with pathologically altered oscillatory activity. While synchronized oscillations between 13 and 30 Hz are increased within a cortico-subcortical network, cortico-muscular coupling (CMC) is decreased. The present study aims at investigating the effect of non-invasive transcranial alternating current stimulation (tACS) of the primary motor cortex (M1) on motor symptoms and motor-cortical oscillations in PD. In 10 PD patients and 10 healthy control subjects, static isometric contraction, dynamic fast finger tapping, and diadochokinesia of the more severely affected hand were investigated prior to and shortly after tACS of the contralateral M1 at 10 Hz vs. 20 Hz vs. sham. During isometric contraction, neuromagnetic activity was recorded using magnetoencephalography. 20 Hz tACS attenuated beta band CMC during isometric contraction and amplitude variability during finger tapping in PD patients but not in healthy control subjects. 10 Hz tACS yielded no significant after-effects. The present data suggest that PD is associated with pathophysiological alterations which abet a higher responsiveness toward frequency-specific tACS – possibly due to pathologically altered motor-cortical oscillatory synchronization at frequencies between 13 and 30 Hz.

## INTRODUCTION

Parkinson’s disease (PD) is a progressive neurodegenerative disorder expressed by bradykinesia, tremor, and rigidity. It is characterized by a degeneration of dopaminergic neurons in the substantia nigra pars compacta and pathologically altered oscillatory synchronization within and between brain regions in the alpha (8–12 Hz), beta (13–30 Hz), and gamma (30–90 Hz) frequency ranges (Brown, 2003; Schnitzler et al., 2006; Weinberger et al., 2009). In more detail, pathologically increased beta band synchronization is frequently found in the basal ganglia circuit in PD patients without medication (Brown et al., 2001b; Foffani et al., 2005; Kuhn et al., 2006; Weinberger et al., 2006). Especially movement slowing such as bradykinesia and rigidity – in contrast to tremor – have been related to increased oscillatory beta band synchronization (Brown and Williams, 2005; Kuhn et al., 2006; Chen et al., 2007; Ray et al., 2008; Zaidel et al., 2009). Moreover, cortico-muscular coupling (CMC) as a neurophysiological marker of functional coupling between the primary motor cortex (M1) and peripheral muscles (Hari and Salenius, 1999) was shown to be decreased in PD (Salenius et al., 2002). In healthy subjects, CMC is prominent in the beta frequency band during weak to medium isometric muscle contraction (Kristeva et al., 2007).

Therapeutic treatment with levodopa or deep brain stimulation (DBS) of the subthalamic nucleus (STN) has been proven to improve motor symptoms and normalize pathologically altered oscillations in PD suggesting that oscillatory patterns are dynamic (Brown et al., 2001a; Marsden et al., 2001; Salenius et al., 2002; Krack et al., 2003; Fogelson et al., 2005; Silberstein et al., 2005; Deuschl et al., 2006; Kuhn et al., 2006; Pollok et al., 2009). These studies highlight the pathophysiological relevance of synchronized oscillatory activity for the origin of motor symptoms in PD.

Cortical brain activity has been shown to be modulated by transcranial alternating current stimulation (tACS; Zaehle et al., 2010; Feurra et al., 2011; Brittain et al., 2013). tACS is supposed to interact with or even entrain spontaneous oscillations in a frequency-specific manner by subthreshold modulation of membrane potentials possibly affecting the likelihood of neuronal firing (Zaehle et al., 2010; Zaghi et al., 2010; Feurra et al., 2011). In healthy subjects, 20 Hz tACS temporarily caused movement slowing (Pogosyan et al., 2009; Wach et al., 2013b). We hypothesize that 20 Hz beta band tACS may go along with increased movement slowing, i.e., bradykinesia in PD patients possibly by entrainment of motor-cortical beta oscillations. Since it is unclear to what extent this behavioral effect may be frequency-specific, the study aims at investigating whether stimulation effects may vary depending on the exact stimulation frequency. In addition, we tried to figure out the effects of tACS on motor-cortical oscillations and CMC. Since previous studies suggest that increased oscillatory activity particularly at beta frequency seems to be related to akinesia (Chen et al., 2007; Weinberger et al., 2009; Jenkinson and Brown, 2011), we hypothesize that 20 Hz tACS may yield movement slowing and decrease of CMC.

## MATERIALS AND METHODS

### PD PATIENTS

The study was approved by the local ethics committee and is in accordance with the Declaration of Helsinki. It was accomplished by a sham-controlled, double-blind within-subject-design with patients’ written informed consent. 10 PD patients [five male; mean age 49.4 ± 3.1 years, mean ± standard error of mean (*SEM*)] were classified between Hoehn & Yahr stage I and II (Hoehn and Yahr, 1967). Mean disease duration was 23.3 ± 6.1 months from firm PD diagnosis and mean age was 47.8 ± 3.2 years at firm PD diagnosis. Motor symptoms were clinically rated by an experienced physician by means of the *Unified Parkinson’s Disease Rating Scale* motor score (UPDRS III). Mean UPDRS III score *ON* medication was 19.8 ± 3.2 (range: 6–30). Motor control of the more severely affected hand and underlying neuromagnetic activity were assessed prior to and shortly (i.e., 5 min) after tACS (10 Hz vs. 20 Hz vs. sham) of the M1 contralateral to the more severely affected hand. Sessions of 10 Hz vs. 20 Hz vs. sham tACS were separated by at least 1 week in order to avoid carry-over effects. In five patients (three male, two female) the right hand was affected more severely and tACS was applied above the contralateral left M1. In five patients (two male, three female) the left hand was affected more seriously and the contralateral right M1 was stimulated. All patients participated *ON* medication to control for floor effects in the recorded parameters. Medical treatment comprised dopamine agonists and MAO-B-inhibitors. Mean equivalent daily L-Dopa dose was 270.9 ± 123.7 mg.

Prior to experimental inclusion, by default patients had undergone a detailed neurological examination in the Department of Neurology, Heinrich-Heine-University including neuropsychological testing and routine laboratory tests. General exclusion criteria were clinically manifest depression or dementia, increased disposition for convulsions and seizures, metal implants, cardiac or brain pacemaker, or other serious neurological, psychiatric or internal diseases.

### CONTROL SUBJECTS

In order to elucidate whether tACS after-effects observed in patients are specific to PD, 10 healthy subjects (five male; mean age 47.8 ± 4.3 years) were included in the study. Control subjects were matched to the patient group with respect to age, gender and performing hand. All subjects provided written informed consent prior to study participation and fulfilled the general inclusion criteria. The control group received 20 Hz tACS only administered in one single session with the same stimulation parameters as PD patients. The order of motor tasks was counterbalanced across subjects.

### DESIGN

**FIGURE 1:**
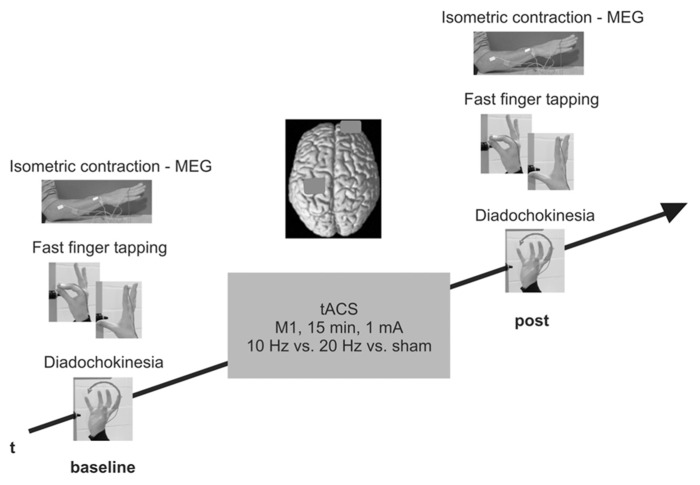
**Experimental design.** Isometric contraction during MEG and fast finger tapping/diadochokinesia were investigated prior to and shortly after tACS. PD patients attended three separate sessions (10 Hz vs. 20 Hz vs. sham), control subjects received 20 Hz tACS only.

Neuromagnetic activity was recorded for 8 min using magnetoencephalography (MEG) during isometric contraction and rest of the more severely affected forearm while subjects were seated in the magnetically shielded room (MSR). Outside the MSR, subjects performed dynamic fast finger tapping and diadochokinesia for 12 s, respectively. Subsequently, subjects received a 15 min tACS of the M1 contralateral to the performing, more severely affected hand outside the MSR. Shortly (i.e., 5 min) after tACS termination, subjects performed the same tasks while neuromagnetic activity and movement characteristics were recorded. Order of tACS (10 Hz vs. 20 Hz vs. sham) and movement tasks was counterbalanced across subjects and sessions but remained constant within one session. The study design is schematically illustrated in **Figure 1**.

### NEUROMAGNETIC RECORDINGS – ISOMETRIC CONTRACTION

Neuromagnetic activity was recorded using a 306 channel whole head MEG system (Elekta Neuromag Oy, Helsinki, Finland) during periods of isometric contraction and relaxation, i.e., rest. To this end, subjects were seated in a MSR while performing the task. The arms rested on a pad fixed to the chair. Immediately before MEG data acquisition individual maximum contraction strength was measured when patients were asked to contract their forearm muscles at their best effort lifting the more severely affected forearm with an angle of 30° with fingers abducted. During neuromagnetic recordings, subjects held the more severely affected forearm for 1 min at this position and at 30% of maximum contraction strength. The elbow remained on the pad. Then the forearm was laid down and relaxed for another minute. The entire task duration was 8 min alternating four times between 1 min of isometric contraction and rest, respectively. Constant isometric contraction was controlled online by means of the electromyogram (EMG) recordings of the extensor digitorum communis muscle (EDC), and the first dorsal interosseus muscle (FDI) of the performing forearm serving as peripheral reference signal for the calculation of CMC. Segments of weaker contraction and movement artifacts were excluded from the analysis after visual offline control. Patients were able to keep contraction strength constant across measurements without fatigue. Eye movements were deduced via a vertical electrooculogram (EOG). All signals were recorded with a sampling frequency of 1000 Hz and filtered with a band pass filter of 0.03–330 Hz. For data analysis, signals of the 204 gradiometers only were selected. Four head position indication (HPI) coils placed on the scalp were localized with respect to three defined anatomical points (nasion and both preauricular points) using a three-dimensional digitizer (Polhemus Isotrak^®^ II, Polhemus Navigation Sciences, Colchester, VT, USA). Prior to each measurement, the head position within the MEG helmet was localized via short electromagnetic signals of the HPI coils.

### BEHAVIORAL RECORDINGS – FAST FINGER TAPPING AND DIADOCHOKINESIA

Outside the MSR subjects performed proximal and distal movements with the more severely affected hand as measured by diadochokinesia and fast finger tapping. Subjects were instructed to perform both tasks as fast as possible for 12 s, respectively. Both tasks were chosen since their execution is known to be impaired in PD patients. Diadochokinesia exemplifies proximal movements and requires subjects to perform revolving pronation and supination of the wrist. Fast finger tapping indicating distal movements was investigated by lifting and tapping the stretched index finger toward the thumb. Movement trajectories were analyzed in three-dimensional space using an ultra sound motion detection system (CMS 10, Zebris Medical, Isny, Germany). To this end, an ultra sound marker was attached dorsally to the index finger at the distal phalanx above the nail. Sampling rate was 200 Hz.

### TRANSCRANIAL ALTERNATING CURRENT STIMULATION

The hand region of M1 contralateral to the more severely affected side was localized by single transcranial magnetic stimulation (TMS) pulses using a standard figure of eight coil (MC-B70; outer winding diameter 80 mm) connected to a MagPro TMS stimulator (Alpine Biomed, Planegg, Germany). The coil was placed tangentially to the scalp with the handle pointing backward and laterally at about 45° away from the midline inducing an initial posterior–anterior current flow in the brain. We first localized the optimal cortical representation of the FDI muscle of the more severely affected side by eliciting motor-evoked potentials (MEP). By moving the coil in 0.5 cm steps anterior, posterior, medial, and lateral to this area, the exact localization of the spot which elicited the maximum FDI motor response was determined as motor hot spot.

Transcranial alternating current stimulation was applied in the standard montage via two saline-soaked sponge electrodes (35 cm^2^) placed on the skull surface (DC-Stimulator Plus, Eldith, NeuroConn, Ilmenau, Germany). The stimulation electrode was attached above the M1 hot spot, while the reference electrode was placed over the orbita contralateral to the stimulation electrode. tACS was applied with 10 Hz vs. 20 Hz vs. sham in three separate sessions for 15 min, respectively (intensity 1 mA (peak-to-peak-amplitude), current density under the electrode 0.0286 mA/cm^2^, sinusoidal waveform). The current was ramped up and down for the first and last 5 s of stimulation. Impedance was kept below 5 kOhm. Stimulation was carried out in accordance with current safety guidelines for electrical current stimulation (Nitsche et al., 2003; Rossi et al., 2009).

For sham stimulation, active tACS was applied only within the first 30 s including ramping up and down for 5 s in order to elicit a short tingling sensation usually perceived at stimulation onset. In half of the subjects, sham stimulation was applied at 10 Hz and at 20 Hz, respectively. Subjects and investigator were blinded with respect to stimulation (10 Hz vs. 20 Hz vs. sham). Since tACS at frequencies roughly between 5 and 50 Hz elicits flicker sensations due to retinal stimulation (Paulus, 2010), a masking flicker stimulus was presented on a screen in front of the subjects using E-Prime^®^ (Psychology Software Tools Inc.) during each stimulation (verum and sham).

## DATA ANALYSIS

### NEUROMAGNETIC RECORDINGS – ISOMETRIC CONTRACTION

Neuromagnetic data were analyzed in sensor space using the FieldTrip toolbox (Oostenveld et al., 2011). The EMG signal of the EDC muscle was segmented with respect to hold (4 × 1 min) and rest (4 × 1 min) periods. The first and last 3 s of each segment were cut from further analysis in order to remove movement-related activation. The EMG signal was high pass filtered at 20 Hz and rectified. MEG data were notch filtered at 50 Hz. Fast Fourier transform (FFT) was applied to transform the data from the time into the frequency domain. FFT sample size was 1024. After applying a Hanning taper for frequencies below 30 Hz and a discrete prolate spheroidal sequence (dpss) taper for frequencies above 30 Hz, cross-spectral density was calculated with a resolution of 0.98 Hz. For frequencies between 30 and 90 Hz smoothing of 2 Hz was conducted.

Frequencies of interest were subdivided into alpha (8–12 Hz), beta (13–30 Hz), low gamma (30–45 Hz), and high gamma (55–90 Hz) bands. These frequencies have been closely related to motor control and have been shown to be altered in PD (for an overview see Schnitzler and Gross, 2005). In the respective frequency ranges, frequencies of maximum power and CMC and the respective amplitudes were determined by detection of the largest peaks in each frequency band in each individual data set. Spectral power estimates synchronization of local oscillatory activity within brain regions. To reduce inter-individual variability power was estimated by logarithmic transformation (Halliday et al., 1995). CMC is an established method for the quantification of functional coupling between M1 and the contralateral peripheral muscle in the frequency domain. Coherence describes linear relationships between EMG and MEG signals normalized between 0% reflecting independence and 100% reflecting complete linear dependence between two signals.

Since individual magnetic resonance imaging (MRI) scans (T1 weighted; 3 Tesla Magnetom®, Siemens, Erlangen, Germany) of six patients and eight control subjects were available, additional source analysis was performed in these data sets. Source analysis was performed with Dynamic Imaging of Coherent Sources (DICS; Gross et al., 2001) using boundary element models (BEM). The EMG served as reference signal for the localization of M1 showing the strongest EMG-M1 coherence. MEG and MRI data were co-registered and aligned by means of the four HPI coil positions and three anatomical landmarks (nasion and both preauricular points).

### BEHAVIORAL DATA – FAST FINGER TAPPING AND DIADOCHOKINESIA

Fast finger tapping and diadochokinesia were analyzed with respect to mean frequency (in Hz), amplitude (in mm) and amplitude variation (in % as mean relative change of amplitude) using the analysis software WinData (Zebris Medical, Isny, Germany). Parameters were calculated as Euclidean norm implementing movement information of all three spatial axes.

The first and last second of each data set was excluded from the analysis leaving continuous data segments of 10 s, respectively.

### STATISTICS

In a first step, data were checked for Gaussian distribution by means of the Kolmogorov–Smirnov-Test. Then repeated measurements analyses of variance (ANOVA) with factors *stimulation* (10 Hz vs. 20 Hz vs. sham) and *time* (baseline vs. post) were performed for frequencies and amplitudes of maximum power and CMC. Greenhouse–Geisser corrected *p*-values are provided when appropriate. To account for intra- and inter-individual variability of (i) frequencies and amplitudes of maximum power and CMC during isometric contraction and during rest and (ii) frequency, amplitude and amplitude variation during fast finger tapping and diadochokinesia, in each session relative changes with respect to baseline were used for statistical comparison. Separate ANOVAs for changes to baseline were calculated with factor *stimulation* (10 Hz vs. 20 Hz vs. sham). *Post hoc* tests were calculated by means of two-tailed *t*-tests. *p*-values were corrected for multiple testing (Holm, 1979). All statistical comparisons were calculated with IBM SPSS Statistics 20.

## RESULTS

### PD PATIENTS

#### Power During Isometric Contraction

Primary motor cortex power was analyzed separately for the alpha, beta, low gamma, and high gamma frequency bands. Prior to stimulation, power peaks ipsilateral to stimulation were found at 10.0 ± 0.4 Hz, 17.2 ± 1.0 Hz, 40.0 ± 1.4 Hz, and 61.0 ± 1.1 Hz. Most discernible M1 power peaks contralateral to stimulation became evident at 9.8 ± 0.4 Hz, 16.6 ± 0.7 Hz, 40.8 ± 1.5 Hz, and 63.0 ± 1.5 Hz. After stimulation, no significant frequency shifts of ipsilateral power [alpha *F*(2,18) = 1.232, *p* = 0.315, beta *F*(2,18) = 1.628, *p* = 0.224, low gamma *F*(2,18) = 0.279, *p* = 0.760, high gamma *F*(2,18) = 1.992, *p* = 0.165] and contralateral power were found [alpha *F*(2,18) = 2.090, *p* = 0.174, beta *F*(2,18) = 0.253, *p* = 0.677, low gamma *F*(2,18) = 0.991, *p* = 0.390, high gamma *F*(2,18) = 0.227, *p* = 0.799].

For relative changes to baseline of power amplitude, no significant stimulation effects on ipsilateral power [alpha *F*(2,29) = 1.045, *p* = 0.366, beta *F*(2,29) = 1.558, *p* = 0.229, low gamma *F*(2,29) = 0.853, *p* = 0.437, high gamma *F*(2,29) = 0.869, *p* = 0.431] and contralateral power were found [alpha *F*(2,29) = 1.597, *p* = 0.221, beta *F*(2,29) = 0.927, *p* = 0.408, low gamma *F*(2,29) = 1.339, *p* = 0.279, high gamma *F*(2,29) = 0.699, *p* = 0.506]. Results are summarized in **Table 1A**.

**Table 1:** Relative changes to baseline in **%** **(A)** of CMC and power (mean ± *SEM*) following tACS during isometric contraction and **(B)** of fast finger tapping and diadochokinesia in PD patients.

	Alpha band	Beta band	Low gamma band	High gamma band
**(A) ISOMETRIC CONTRACTION**
**Contralateral CMC**
10 Hz	112.10 ± 7.15	103.60 ± 4.56	105.81 ± 8.98	105.44 ± 2.86
20 Hz	102.39 ± 9.54	**89.68 ± 3.97**	100.10 ± 3.89	96.82 ± 3.99
Sham	100.52 ± 6.82	102.06 ± 3.40	96.73 ± 5.71	105.28 ± 3.52
*Stimulation*	*F*(2,29) = 0.62, *p* = 0.548	*F*(2,29) = 3.63, ***p* = 0.040**	*F*(2,29) = 0.49, *p* = 0.617	*F*(2,29) = 1.99, *p* = 0.156
**Contralateral power**
10 Hz	92.39 ± 12.69	89.31 ± 6.07	89.56 ± 5.98	91.24 ± 5.63
20 Hz	104.31 ± 9.49	98.16 ± 10.34	98.86 ± 2.44	99.88 ± 1.50
Sham	122.26 ± 13.18	131.13 ± 37.79	99.23 ± 5.06	93.36 ± 7.29
*Stimulation*	*F*(2,29) = 1.60, *p* = 0.221	*F*(2,29) = 0.93, *p* = 0.408	*F*(2,29) = 1.34, *p* = 0.279	*F*(2,29) = 0.70, *p* = 0.506
**Ipsilateral power**
10 Hz	89.43 ± 12.13	89.43 ± 6.82	93.63 ± 3.77	96.20 ± 2.51
20 Hz	112.01 ± 10.92	95.38 ± 13.73	102.29 ± 6.08	100.22 ± 3.14
Sham	109.13 ± 12.95	119.04 ± 15.40	100.44 ± 8.74	97.36 ± 5.80
*Stimulation*	*F*(2,29) = 1.05, *p* = 0.366	*F*(2,29) = 1.56, *p* = 0.229	*F*(2,29) = 0.85, *p* = 0.437	*F*(2,29) = 0.87, *p* = 0.431

	**Frequency**	**Amplitude**	**Amplitude variation**	

**(B) FAST FINGER TAPPING**
10 Hz	106.01 ± 7.03	99.13 ± 4.95	109.06 ± 17.35	
20 Hz	96.27 ± 5.46	98.43 ± 4.44	**69.21 ± 7.03**	
Sham	113.16 ± 5.56	99.05 ± 2.09	133.27 ± 14.18	
*Stimulation*	*F*(2,29) = 1.96, *p* = 0.161	*F*(2,29) = 0.01, *p* = 0.991	*F*(2,29) = 5.69, ***p* = 0.009**

**DIADOCHOKINESIA**
10 Hz	100.36 ± 8.24	102.11 ± 10.34	92.77 ± 12.43
20 Hz	95.24 ± 6.57	96.48 ± 4.42	99.33 ± 6.96
Sham	103.74 ± 5.62	102.44 ± 5.11	107.86 ± 12.84
*Stimulation*	*F*(2,29) = 0.38, *p* = 0.685	*F*(2,29) = 0.22, *p* = 0.803	*F*(2,29) = 0.47, *p* = 0.632

*ANOVAs with factor *stimulation* (10 Hz vs. 20 Hz vs. sham) were performed. Significant changes are highlighted.*

#### Cortico-Muscular Coherence During Isometric Contraction

**FIGURE 2:**
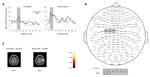
**Beta band cortico-muscular coupling (CMC) during isometric contraction. (A)** Most distinct beta band CMC peaks between S1/M1 sensors and contralateral EDC muscle before and after 20 Hz tACS in one representative patient (left panel) and control subject (right panel). **(B)** One representative MEG sensor plot within the frequency range from 10 Hz to 30 Hz. The most prominent beta band CMC peaks are localized in sensors corresponding to the left S1/M1 region. **(C)** Beta band CMC source localization in Brodmann Area (BA) 3/4 in two representative individual data sets.

CMC was analyzed separately for the alpha, beta, low gamma, and high gamma frequency bands. The most discernible peak was determined in each frequency band, respectively. Prior to stimulation, CMC peaks were found at 9.6 ± 0.2 Hz, 18.2 ± 0.7 Hz, 36.4 ± 0.7 Hz, and 73.1 ± 2.1 Hz. Exemplarily for beta band CMC during isometric contraction, pre-post comparison, and topographical distribution across MEG sensors are illustrated in **Figures 2A,B**. These peaks were localized within the primary sensorimotor cortex (S1/M1) contralateral to the more severely affected forearm corresponding to Brodmann Area 3/4 (**Figure 2C**). After stimulation (10 Hz vs. 20 Hz vs. sham), no significant frequency shift of CMC was found [alpha *F*(2,18) = 2.793, *p* = 0.088, beta *F*(2,18) = 1.196, *p* = 0.325, low gamma *F*(2,18) = 0.790, *p* = 0.469, high gamma *F*(2,18) = 0.065, *p* = 0.937].

**FIGURE 3:**
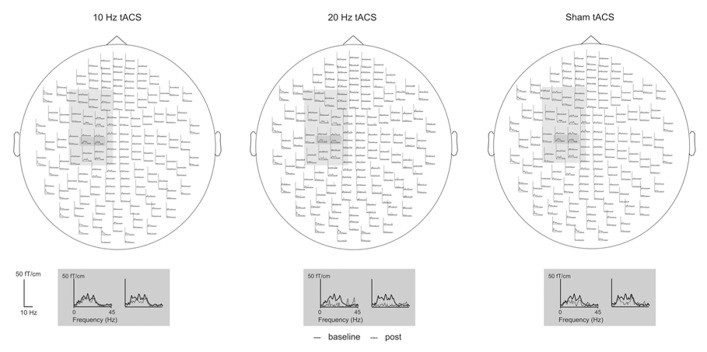
**Mean modulation of beta band CMC amplitude in PD patients following 10 Hz, 20 Hz, and sham tACS.** Patients performed the isometric contraction with the more severely affected right forearm. Baseline is averaged across all sessions. The sensors selected for the analysis are highlighted in light gray. Sensors with the most prominent modulation following 20 Hz tACS are enlarged.

**FIGURE 4:**
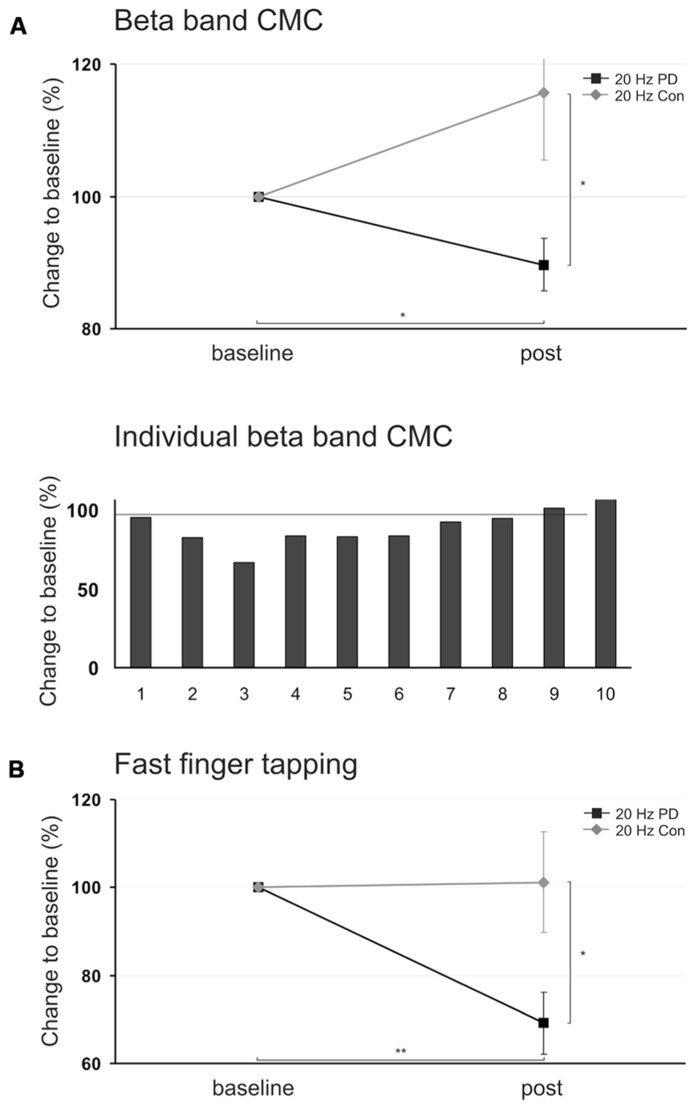
**(A)** Upper panel: Mean relative changes of beta band cortico-muscular coupling (CMC) amplitude during isometric contraction in PD patients and healthy control subjects following 20 Hz tACS (mean ± *SEM*), lower panel: Individual relative changes of beta band CMC amplitude in 10 PD patients following 20 Hz tACS. **(B)** Relative changes of amplitude variation during fast finger tapping following 20 Hz tACS in PD patients and healthy control subjects. Patients performed the tasks with the more severely affected forearm, control subjects were matched, respectively. Asterisk indicates *p* < 0.05, two asterisks indicate *p* < 0.01.

For CMC amplitude changes during periods of isometric contraction at beta frequency, ANOVA revealed a significant main effect of factor *stimulation* [*F*(2,29) = 3.628, *p* = 0.040]. *Post hoc* *t*-tests revealed that 20 Hz tACS significantly reduced beta band CMC amplitude as compared to sham stimulation [20 Hz vs. sham: *t*(9) = -3.286, *p* = 0.018] and to 10 Hz tACS [10 Hz vs. 20 Hz: *t*(9) = 3.469, *p* = 0.021] whereas 10 Hz tACS effects were not significantly different from sham stimulation [10 Hz vs. sham: *t*(9) = 0.428, *p* = 0.678]. One-sample *t*-test, furthermore, indicated that the beta band CMC amplitude due to 20 Hz tACS was significantly reduced as compared to baseline level [20 Hz vs. 100: *t*(9) = -2.598, *p* = 0.029; **Figure 4A**]. Modulation of absolute beta band CMC amplitudes during isometric contraction due to tACS across MEG sensors is illustrated in **Figure 3**.

ANOVAs for relative CMC amplitude changes within the alpha [*F*(2, 29) = 0.615, *p* = 0.548], low gamma [*F*(2,29) = 0.492, *p* = 0.617], and high gamma frequency bands [*F*(2,29) = 1.993, *p* = 0.156] did not show any significant main effects of factor *stimulation*. Results are summarized in **Table 1A**. Accordingly, no significant amplitude modulation as compared to baseline level was evident following 10 Hz, 20 Hz, and sham tACS [one-sample *t*-test: alpha: 10 Hz vs. 100: *t*(9) = 1.693, *p* = 0.125; 20 Hz vs. 100: *t*(9) = 0.251, *p* = 0.807; sham vs. 100: *t*(9) = 0.076, *p* = 0.941; low gamma: 10 Hz vs. 100: *t*(9) = 0.647, *p* = 0.534; 20 Hz vs. 100: *t*(9) = 0.027, *p* = 0.979; sham vs. 100: *t*(9) = -0.573, *p* = 0.581; high gamma: 10 Hz vs. 100: *t*(9) = 1.902, *p* = 0.090; 20 Hz vs. 100: *t*(9) = -0.795, *p* = 0.447; sham vs. 100: *t*(9) = 1.497, *p* = 0.169].

#### Power During Rest

For relative M1 power amplitude changes during rest, separate ANOVAs for each frequency band with factor *stimulation* were performed. No significant stimulation effects on ipsilateral [alpha *F*(2,29) = 0.359, *p* = 0.702, beta *F*(2,29) = 2.420, *p* = 0.108, low gamma *F*(2,29) = 1.465, *p* = 0.249, high gamma *F*(2,29) = 0.243, *p* = 0.786], and contralateral M1 power amplitudes [alpha *F*(2,29) = 1.583, *p* = 0.224, beta *F*(2,29) = 1.591, *p* = 0.222, low gamma *F*(2,29) = 2.761, *p* = 0.081, high gamma *F*(2,29) = 0.277, *p* = 0.760] were found.

#### Cortico-Muscular Coherence During Rest

For CMC during rest, separate ANOVAs for each frequency band with factor *stimulation* were performed. No significant stimulation effects on relative CMC amplitude changes were found [alpha *F*(2,29) = 0.447, *p* = 0.644, beta *F*(2,29) = 0.138, *p* = 0.872, low gamma *F*(2,29) = 0.085, *p* = 0.919, high gamma *F*(2,29) = 0.397, *p* = 0.676].

#### Fast Finger Tapping and Diadochokinesia

Relative changes of movement trajectories of the more severely affected hand were analyzed with respect to frequency, amplitude, and amplitude variation separately for fast finger tapping and diadochokinesia. ANOVA for amplitude variation during fast finger tapping revealed a significant main effect of factor *stimulation* [*F*(2,29) = 5.694, *p* = 0.009]. *Post hoc* paired *t*-tests showed that 20 Hz tACS yielded less amplitude variation during fast finger tapping as compared to sham stimulation [20 Hz vs. sham: *t*(9) = -3.910, *p* = 0.012] – indicating more regular motor performance following 20 Hz tACS. One-sample *t*-test, furthermore, indicated that finger tapping amplitude variation due to 20 Hz tACS was significantly reduced as compared to baseline level [20 Hz vs. 100: *t*(9) = -4.379, *p* = 0.002, **Figure 4B**].

10 Hz tACS effects did not differ significantly from sham stimulation [10 Hz vs. sham: *t*(9) = -0.920, *p* = 0.381] and 20 Hz tACS [10 Hz vs. 20 Hz: *t*(9) = 2.313, *p* = 0.092].

Finger tapping frequency [*F*(2,29) = 1.955, *p* = 0.161] and amplitude [*F*(2,29) = 0.009, *p* = 0.991] were not significantly influenced by tACS. For diadochokinesia, no significant effects were found [amplitude variation *F*(2,29) = 0.467, *p* = 0.632, frequency *F*(2,29) = 0.384, *p* = 0.685, amplitude *F*(2,29) = 0.221, *p* = 0.803]. Results are summarized in **Table 1B**.

### CONTROL SUBJECTS

Data analysis was limited to beta band CMC amplitude during isometric contraction and to amplitude variation during fast finger tapping, since these parameters were found to be significantly affected by tACS in PD patients.

#### Cortico-Muscular Coherence During Isometric Contraction

Baseline CMC amplitude at beta frequency did not differ significantly between PD patients *ON* regular medication (13.9 ± 1.7%) and controls [14.9 ± 2.5%; independent sample *t*-test: *t*(18) = -0.338, *p* = 0.739], but the most discernible beta band CMC peaks were found at 18.3 ± 1.0 Hz in PD patients and at 23.2 ± 1.6 Hz in controls [*t*(18) = -2.594, *p* = 0.018]. Relative changes of CMC amplitude were analyzed using paired *t*-test. 20 Hz tACS did not yield a significant beta band CMC modulation when compared to baseline [*t*(9) = -1.548, *p* = 0.156, **Figure 4A**].

Relative changes of beta band CMC amplitude after 20 Hz tACS led toward opposing directions comparing PD patients (89.7 ± 4.0% post) and healthy subjects (115.6 ± 10.1% post) and were statistically significant [independent sample *t*-test: *t*(18) = -2.391, *p* = 0.028, **Figure 4A**].

#### Fast Finger Tapping and Diadochokinesia

Amplitude variation during finger tapping was not significantly affected by 20 Hz tACS in controls [paired t-test: *t*(9) = 0.751, *p* = 0.472, **Figure 4B**]. Baseline amplitude variation did not differ between PD patients and controls [independent sample *t*-test: *t*(18) = 0.778, *p* = 0.447]. But, 20 Hz tACS yielded significantly less amplitude variation during finger tapping in PD patients (69.2 ± 7.0% post) as compared to healthy subjects [101.2 ± 11.4% post; independent sample *t*-test: *t*(18) = -2.381, *p* = 0.028, **Figure 4B**].

## DISCUSSION

Intention of the present study was to investigate frequency-specific after-effects of tACS on motor performance and the underlying motor-cortical oscillations in PD patients. The study aimed at elucidating to what extent behavioral motor changes (i) may vary depending on stimulation frequency and (ii) may rely on neurophysiological changes like motor-cortical oscillatory activity and CMC. We hypothesized that predominant motor-cortical beta oscillations induced by 20 Hz tACS may go along with movement slowing.

After-effects of tACS at 10 Hz and 20 Hz over M1 were measured during isometric contraction, fast finger tapping, and diadochokinesia of the more severely impaired forearm in PD patients and matched controls. Neuromagnetic activity was recorded during isometric contraction, and dynamic movement trajectories were analyzed during fast finger tapping and diadochokinesia. While no significant after-effects on local oscillatory activity by means of M1 power within the alpha, beta, or gamma frequency bands became evident, 20 Hz tACS yielded significantly reduced beta band CMC amplitude during isometric contraction in PD patients but not in healthy control subjects. Performance of the dynamic motor tasks, furthermore, revealed significantly reduced amplitude variation during fast finger tapping after 20 Hz tACS in PD patients only.

### EFFECTS OF tACS ON BETA BAND CMC

CMC is a neurophysiological marker of functional coupling between M1 and contralateral active muscles most evident in the beta frequency band between 13 and 30 Hz during static motor control such as isometric contraction (Salenius and Hari, 2003; Kristeva et al., 2007). It represents an established measure of the integrity of the pyramidal system (Mima and Hallett, 1999) and is assumed to promote effective corticospinal interaction in favor of static motor control (Kristeva et al., 2007).

A characteristic feature of PD patients *OFF* medication is decreased beta band CMC amplitude which is normalized following L-Dopa intake (Salenius et al., 2002). Decreased CMC amplitude has been related to reduced high frequency cortically generated drives to contralateral muscles affecting ongoing motor control mechanisms contributing to PD motor symptoms (Volkmann, 1998; Marsden et al., 2001). The present data show a decrease in beta band CMC amplitude in PD patients following 20 Hz tACS only. One may assume a selective inhibition of the cortical drive to muscles in PD patients after 20 Hz tACS by either (i) directly affecting the connectivity between M1 and the contralateral active muscles, or by (ii) local cortical oscillatory entrainment during frequency-specific stimulation (Zaehle et al., 2010; Neuling et al., 2012, 2013) promoting 20 Hz oscillations which then may (indirectly) affect the cortico-muscular drive. Since offline no significant local M1 power changes were observed, one may further speculate that (iii) oscillatory entrainment might have spread to subcortical structures – possibly outlasting stimulation cessation – which was not captured by the present MEG analyses. The present data suggest that in PD patients tACS attenuates beta band CMC possibly due to neuroplastic alterations within the motor system outlasting the time period of stimulation – in accordance with a cross-frequency after-effect of tACS on CMC but not local M1 power previously shown using MEG (Wach et al., 2013a). Due to the study design we cannot rule out the possibility that tACS may have induced cortical entrainment during stimulation but it seems rather unlikely that such online entrainment accounts for offline effects on CMC observed here. Evidence exists that with prolonged stimulation duration the neurophysiological mechanisms underlying the after-effects of non-invasive brain stimulation may be associated with neuroplastic alterations rather than oscillatory entrainment (Thut et al., 2011; Antal and Paulus, 2013; Herrmann et al., 2013). We here would like to stress that we do not rule out entrainment but would like to argue in favor of (i) neuroplastic changes yielding after-effects on CMC or (ii) spreading of oscillatory entrainment to brain areas remote from M1. Interestingly enough, this effect was found in PD patients but not in healthy controls.

Since CMC amplitude has been shown to be decreased in PD patients due to dopaminergic depletion (Salenius et al., 2002), the present results suggest that tACS after-effects vary with the dopaminergic state supporting previous data (Monte-Silva et al., 2009, 2010). Thus, the present data suggest that PD may be associated with neuropathological prerequisites promoting higher responsiveness toward tACS. The effect of tACS on CMC was found following 20 Hz stimulation only supporting a specific significance of beta oscillations for the occurrence of neurophysiological markers of PD like reduced CMC.

Data from a recent study suggest state-dependent differences of tACS effects (Neuling et al., 2013) suggesting that the efficacy of stimulation and direction of after-effects depend on the concordance between ongoing brain oscillations and stimulation frequency. Along this line, one might argue that application of tACS during isometric contraction and *not* during rest might have yielded stronger after-effects on neurophysiological measures. Nevertheless, this potential limitation of the present study cannot account for the different effects in PD patients and control subjects. Recently, individualized frequency and timing of tACS have been shown to suppress the resting tremor amplitude in tremor-dominant PD patients (Brittain et al., 2013) providing clinical implications of tACS protocols. Since the most prominent beta band CMC peak was at a significantly higher frequency in controls as compared to PD patients, one might argue that tACS at individual CMC frequencies would have been more effective. But, again, different effects on CMC in patients and controls are not likely to be explained by a lack of stimulation at individual CMC frequencies.

### EFFECTS OF tACS ON FAST FINGER TAPPING VARIABILITY

Clinical assessment of PD is standardized by means of the UPDRS. Dynamic motor tasks like fast finger tapping and diadochokinesia manifest reduction of movement speed, i.e., bradykinesia in these patients. The tasks may rely on different motor control mechanisms since finger tapping is assumed to involve distal and diadochokinesia rather proximal movements (Timmermann et al., 2008). The present results demonstrate an effect of 20 Hz tACS on motor variability in PD patients during fast finger tapping but not diadochokinesia suggesting a particular impact on distal, finely tuned motor control in PD patients. During the finger tapping task, subjects lifted and tapped the stretched index finger toward the thumb with the largest amplitude and fastest rate. Reduced amplitude variability following 20 Hz tACS manifests rather stable movement execution of the more severely affected hand – corroborating the hypothesis that beta oscillations may promote the maintenance of the current motor set (Engel and Fries, 2010). This result, furthermore, reveals a piece of evidence that PD patients were able to perform more fluid finger tapping following 20 Hz tACS – a finding which might imply a beneficial impact on distal motor control mechanisms.

Interestingly enough and in contrast to the initial hypothesis of movement slowing, in both tasks movement frequency was not significantly reduced due to 20 Hz tACS. Movement slowing has previously been shown in healthy subjects during (Pogosyan et al., 2009) and after 20 Hz tACS of M1 (Wach et al., 2013b). A possible assumption to account for the discrepant effect may be the higher age of subjects in the present study. Additionally, the time between stimulation cessation and motor tasks was longer in the present study as compared to Wach et al. (2013b) due to technical restrictions. Thus, although speculative at the moment, we cannot rule out the possibility that relatively small behavioral effects might have been evident directly after stimulation but may have already vanished at the time of post measurement.

Since the observed effects were not evident in healthy control subjects, we conclude that pathologically altered beta band synchronization in PD may promote a higher responsiveness toward tACS effects at this frequency. These results corroborate the assumption of reciprocal interaction between motor symptoms, dopaminergic depletion, and altered central beta oscillations in PD (Jenkinson and Brown, 2011).

## CONCLUSION

The present data show decreased beta band CMC and variability of fast distal movements due to motor-cortical tACS at 20 Hz in PD patients but not in healthy controls. The results suggest that PD is associated with neuropathophysiological alterations which abet a higher responsiveness toward frequency-specific tACS – possibly due to altered PD-related motor-cortical oscillatory synchronization at beta frequency.

## AUTHOR CONTRIBUTIONS

Vanessa Krause: conception and design of the experiment, data collection and analysis, interpretation of the data, drafting the article; Claudia Wach: data collection and analysis, interpretation of the data, critical revision of the article; Martin Südmeyer: interpretation of the data, critical revision of the article; Stefano Ferrea: data collection, interpretation of the data, critical revision of the article; Alfons Schnitzler: interpretation of the data, critical revision of the article; Bettina Pollok: conception and design of the experiment, interpretation of the data, drafting the article.

## Conflict of Interest Statement

The authors declare that the research was conducted in the absence of any commercial or financial relationships that could be construed as a potential conflict of interest.
